# Diabetic Macular Edema Detection Using End-to-End Deep Fusion Model and Anatomical Landmark Visualization on an Edge Computing Device

**DOI:** 10.3389/fmed.2022.851644

**Published:** 2022-04-04

**Authors:** Ting-Yuan Wang, Yi-Hao Chen, Jiann-Torng Chen, Jung-Tzu Liu, Po-Yi Wu, Sung-Yen Chang, Ya-Wen Lee, Kuo-Chen Su, Ching-Long Chen

**Affiliations:** ^1^Information and Communications Research Laboratories, Industrial Technology Research Institute, Hsinchu, Taiwan; ^2^Department of Ophthalmology, Tri-Service General Hospital, National Defense Medical Center, Taipei, Taiwan; ^3^Department of Optometry, Chung Shan Medical University, Taichung, Taiwan

**Keywords:** diabetic macular edema, hard exudate, optic disc and macula, deep learning, visualization

## Abstract

**Purpose:**

Diabetic macular edema (DME) is a common cause of vision impairment and blindness in patients with diabetes. However, vision loss can be prevented by regular eye examinations during primary care. This study aimed to design an artificial intelligence (AI) system to facilitate ophthalmology referrals by physicians.

**Methods:**

We developed an end-to-end deep fusion model for DME classification and hard exudate (HE) detection. Based on the architecture of fusion model, we also applied a dual model which included an independent classifier and object detector to perform these two tasks separately. We used 35,001 annotated fundus images from three hospitals between 2007 and 2018 in Taiwan to create a private dataset. The Private dataset, Messidor-1 and Messidor-2 were used to assess the performance of the fusion model for DME classification and HE detection. A second object detector was trained to identify anatomical landmarks (optic disc and macula). We integrated the fusion model and the anatomical landmark detector, and evaluated their performance on an edge device, a device with limited compute resources.

**Results:**

For DME classification of our private testing dataset, Messidor-1 and Messidor-2, the area under the receiver operating characteristic curve (AUC) for the fusion model had values of 98.1, 95.2, and 95.8%, the sensitivities were 96.4, 88.7, and 87.4%, the specificities were 90.1, 90.2, and 90.2%, and the accuracies were 90.8, 90.0, and 89.9%, respectively. In addition, the AUC was not significantly different for the fusion and dual models for the three datasets (*p* = 0.743, 0.942, and 0.114, respectively). For HE detection, the fusion model achieved a sensitivity of 79.5%, a specificity of 87.7%, and an accuracy of 86.3% using our private testing dataset. The sensitivity of the fusion model was higher than that of the dual model (*p* = 0.048). For optic disc and macula detection, the second object detector achieved accuracies of 98.4% (optic disc) and 99.3% (macula). The fusion model and the anatomical landmark detector can be deployed on a portable edge device.

**Conclusion:**

This portable AI system exhibited excellent performance for the classification of DME, and the visualization of HE and anatomical locations. It facilitates interpretability and can serve as a clinical reference for physicians. Clinically, this system could be applied to diabetic eye screening to improve the interpretation of fundus imaging in patients with DME.

## Introduction

Diabetes is a prevalent disease that affects ~476 million people worldwide (1). Diabetic macular edema (DME), characterized by the accumulation of extracellular fluid that leaks from blood vessels in the macula (2), is one of the complications of diabetes mellitus. DME can appear at any stage of diabetic retinopathy (DR) and is the leading cause of severe vision loss in working-age adults with diabetic mellitus (3). The Early Treatment of Diabetic Retinopathy Study (ETDRS) defined the criteria for DME and demonstrated the benefits of laser photocoagulation therapy (4). Currently, with the revolutionary development of intraocular medication, intravitreal injections of anti-vascular endothelial growth factor (anti-VEGF) and steroid agents are the first-line treatment as alternatives to traditional laser photocoagulation as they provide better vision recovery in patients with center-involved macular edema (5–7).

Early diagnosis plays an important role in DME treatment. Moreover, early management such as intensive diabetes control may reduce the risk of progressive retinopathy (8). Early diagnosis and preemptive treatment are facilitated by frequent diabetic eye screening, which reduces the risk of progression to blindness, and the associated socioeconomic burden. To date, owing to developments in the field of ophthalmic imaging, the detection of DME using optical coherence tomography (OCT) imaging is the gold standard in the decision-making process for DME treatment (9). However, limited by various factors, such as the requirements of expensive equipment and highly specialized technicians, OCT imaging is typically readily available in high-income countries. In contrast, retinal photography examination is feasible and affordable in low-income countries and remote areas (10). However, the number of people with diabetes worldwide is increasing yearly and is estimated to reach 571 million by 2025 (1). The rapid growth of diabetic patients is expected to increase the diagnostic burden associated with DME detection. As such, an efficacious and accurate automatic fundus imaging interpretation system is urgently needed.

In the past decade, several studies have focused on DME detection using feature engineering techniques, which extract features by selecting or transforming raw data. Among them, Siddalingaswamy et al. (11) identified DME by detecting hard exudates (HE) and the macula. Subsequently, decisions were made based on the distance between the HE and the macula. Machine learning algorithms have also been applied in several studies for feature extraction in DME classification (12–15). The advantage of feature engineering is that it utilizes a smaller training dataset to achieve satisfactory performance. However, the identification of salient and useful features depends on the experience of clinicians and is thus subjective and limited. In contrast to feature engineering techniques, deep learning, particularly convolutional neural networks (CNNs), is gaining popularity and has achieved significant success in medical imaging applications. This approach can automatically learn feature extraction by using a backbone network mainly comprising convolutional and pooling layers. Several studies have shown that various architectures of CNN can be used to effectively extract features in fundus images for subsequent classification of DR or DME (16–21).

Moreover, given that deep learning models lack interpretability and are viewed as black boxes (22), visualization of the lesion in fundus images is an important issue. Lesion visualization can improve the interpretability of non-ophthalmologist physicians. In addition, visualization is useful to physicians during an initial assessment before a patient is referred to an ophthalmologist for further evaluation, thereby substantially increasing the screening rate and reducing the workload of ophthalmologists. In addition, lesion visualization could help physicians to monitor the status and progression of the disease.

Generally, deep learning models are implemented in cloud computing environments or high-end computers, which provide more computing power and memory space. However, this is usually expensive and requires considerable network resources. These factors limit the application of deep learning models for medical image analysis in remote or resource-limited areas. Thus, an edge device is potentially suitable for the application of deep learning models for medical image analysis in these areas. Previous studies have demonstrated the feasibility of deploying deep learning models for medical image analysis on edge devices (23–25). However, a system with multiple models for disease classification and visualization requires more computing power and memory. Thus, the implementation of such a system on an edge device is challenging.

In this study, we designed an end-to-end deep fusion network model to perform two deep learning tasks, one for the classification of DME and the other for the visualization of HE lesions. We used a private dataset and two open datasets to evaluate the performance of this fusion model. We also added a second object detector model to identify anatomical landmarks (optic disc and macula). These models were deployed on an edge device. The private dataset was used to assess the performance of the models. Overall, this system could be used for diabetic eye screening by non-specialist physicians or in remote or resource-limited areas to improve the early diagnosis of DME. As a result, diabetic patients may be referred for early assessment and appropriate treatment, which should lead to better outcomes.

## Materials and Methods

### Private Dataset

**Figure 1:**
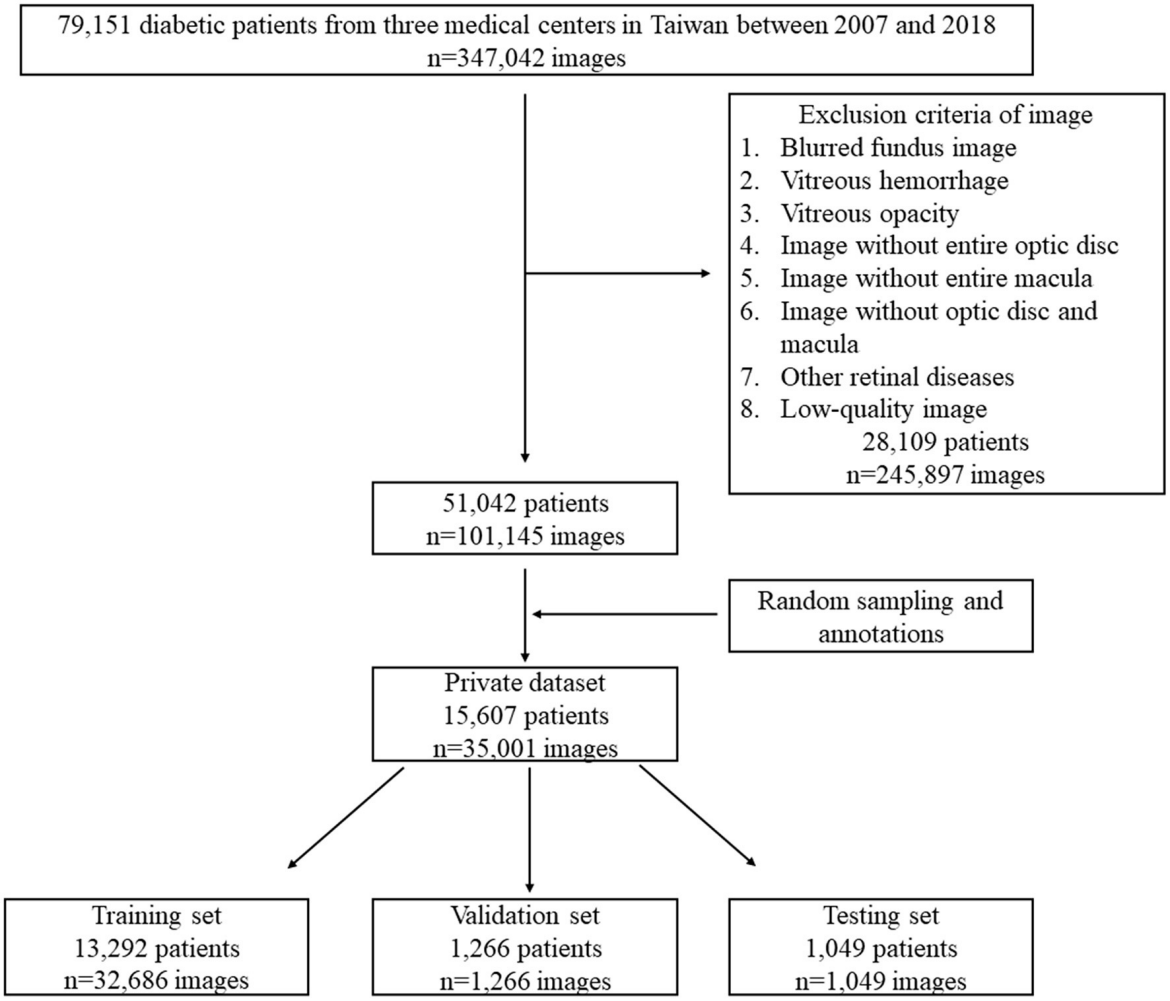
The flowchart of our private dataset.

We enrolled patients who had a diagnosis of diabetic mellitus according to the ICD-9 codes 250.xx or ICD-10 codes E10-E14 between 2007 and 2018 from three medical centers in Taiwan. Patients younger than 20 years of age and with unknown sex were excluded. The retinal photographs were acquired from ZEISS (VISUCAM 200), Nidek (AFC-330), and Canon (CF-1, CR-DGI, CR2, or CR2-AF) fundus cameras with a 45° field-of-view (FOV) and anonymized owing to the retrospective nature of the study. We collected 347,042 fundus images from 79,151 diabetic patients. For the present study, we included image with optic disc and macula to develop models. Blurred fundus image, vitreous hemorrhage, vitreous opacity, image without entire optic disc, image without entire macula, image without optic disc and macula, other retinal diseases, and low-quality image were excluded, and 101,145 fundus images from 51,042 diabetic patients were left for random sampling and annotations. Finally, 35,001 fundus images from 15,607 patients formed our private dataset for model development (The flowchart shown in Figure 1). On our private dataset, the mean age of patients was 57.6 ± 11.8 years and 54.5% were males and 45.5% were females. Eight thousand four hundred and ninety-six patients took only one image and 7,111 patients took more than one image from each eye. The original dimension of the images were 522,728 pixels (724 × 722) to 12,212,224 pixels (4,288 × 2,848). All images were the JPG image format.

### Ethical Considerations

The study was reviewed and approved by the institutional review board (IRB) of the three medical centers: Tri-Service General Hospital (IRB: 1-107-05-039), Chung Shan Medical University Hospital (IRB: CSH: CS18087), and China Medical Hospital (IRB: CMUH10FREC3-062). Given that the identities of all patients in three medical centers were encrypted before fundus images were released, the requirement for signed informed consent of the included patients was waived.

### Annotations of Private Dataset

#### Annotating DME Classification for Fundus Image

We recruited 38 ophthalmologists to annotate the fundus images. Each fundus image was annotated by a group of three ophthalmologists. According to the criteria of ETDRS, DME was defined as any HE at or within 1 disc diameter (1DD) of the center of the macula (4). Each ophthalmologist annotated images by using our annotation tool. We used the majority decision of the three ophthalmologists as the ground truth (GT) of the fundus images. Further, the dataset was split into training, validation, and testing sets by patient level to prevent the same patient in different sets (Figure 1). Eight thousand four hundred and ninety-six of 15,607 patients took only one image and were randomly sampled to validation set (1,266 patients, 1,266 images) and testing set (1,049 patients, 1,049 images). The rest of these patients and 7,111 of 15,607 patients were reserved as a training set (13,292 patients, 32,686 images). Table 1 lists the DME and non-DME profiles of these three subsets.

**Table 1:** Dataset profile for the classification task in the private dataset.

	**Training set**		**Validation set**		**Testing set**	
**Class**	**Number of images**	**Number of patients**	**Number of images**	**Number of patients**	**Number of images**	**Number of patients**
Non-DME	18,921 (57.89%)	10,313	1,140 (90.05%)	1,140	939 (89.51%)	939
DME	13,765 (42.11%)	5,955	126 (9.95%)	126	110 (10.49%)	110
Total	32,686 (100.00%)	16,268	1,266 (100.00%)	1,266	1,049 (100.00%)	1,049

#### Annotating HE Lesions in Fundus Image

**Figure 2:**
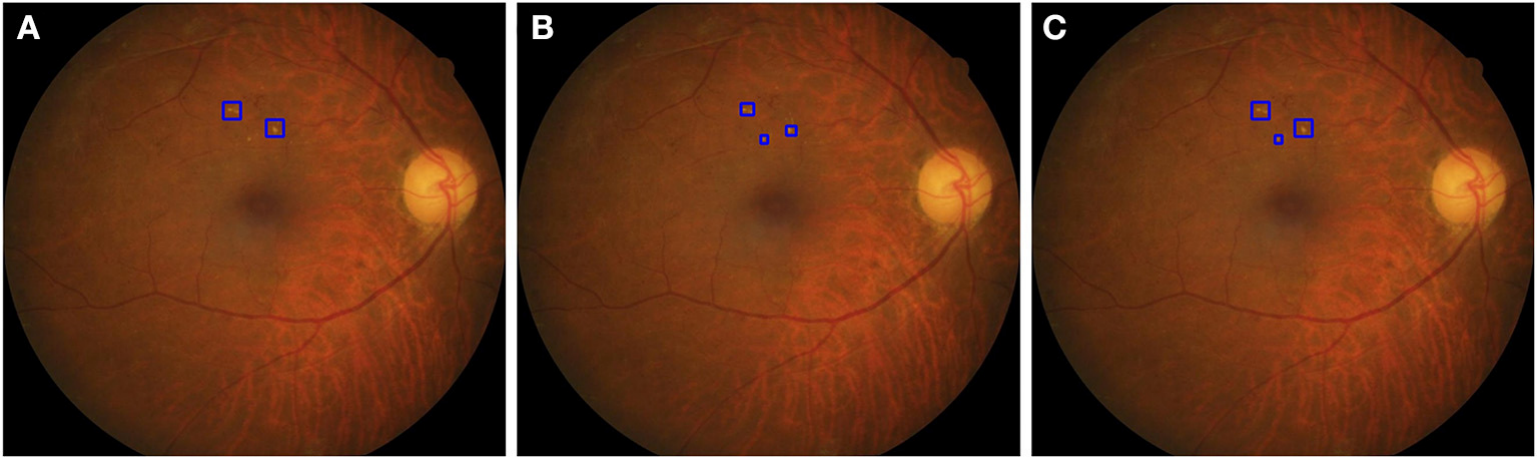
The strategy to obtain a ground truth image. **(A)** The two boxes were annotated by first ophthalmologist. **(B)** The three boxes were annotated by second ophthalmologist. In step 1, the IoU of the top two bounding boxes in **(A,B)** were larger than 0.15, then two larger areas were taken as the GT. In step 2, the IoU of bottom boxes in **(A,B)** were <0.15, the area annotated by the second ophthalmologist was retained as the GT. After step 1 and 2, we obtained a GT image **(C)**.

The HE lesions in each fundus image were also annotated by a group of three ophthalmologists (randomly chosen from 38 ophthalmologists) using a bounding box format. However, three resulting annotations may be different from each other in the number, size, and location of the boxes. We adopted the following procedure to obtain a final GT image for training purposes: (Step 1) The bounding boxes for the image labeled by two ophthalmologists were compared. If an HE lesion was annotated and the intersection over union (IoU) > 0.15, then a larger annotated area was taken as the GT; (Step 2) The bounding boxes of an image labeled by two ophthalmologists were compared. If the HE lesion was annotated and the IoU ≤ 0.15, then both bounding boxes were retained as the GT. After step 1 and 2, we obtained the first GT image as shown in Figure 2. Step 3: First GT image was compared with the image labeled by the third ophthalmologist according to the same method in steps 1 and 2. Then, we obtained the final GT image. In this study, ophthalmologists used bounding boxes to annotate HE lesions in fundus image. The size of the annotated bounding boxes in original images were 9–5,196,672 pixels (9,791.57 ± 36,966.28 pixels). After resized the image, the size of the annotated bounding boxes in model's input images were 1.50–190,008.85 pixels (1,002.99 ± 2,719.79 pixels). However, the annotated bounding boxes only indicated whether existed HE lesions and location, not represented the true size of HE lesions. Therefore, the size of the bounding boxes was usually larger than the true size of the HE lesions. In addition, the profiles of HE labels of the three subsets are shown in Table 2.

**Table 2:** The number of images were annotated HE lesions by ophthalmologists in the private dataset.

	**Training set**	**Validation set**	**Testing set**
Number of images with HE	22,108	583	365
Total	32,686	1,266	1,049

### Open Datasets

Two open datasets were used to evaluate the performance and ability of the proposed model to adapt to different datasets.

#### Messidor-1

The Messidor-1 (26) dataset contained 1,200 fundus images from three ophthalmologic departments in France and was annotated with DR and the risk of DME. All images were acquired using a Topcon TRC NW6 non-mydriatic retinal camera with a 45° FOV. Our grading scheme was slightly different from that of Messidor-1, in which DME was graded according to three categories, with 0, 1, 2 representing “no visible HE,” “HE presence at least 1DD away from the macula,” and “HE presence within 1DD from the macula,” respectively. As previously indicated, HE that occurs within 1DD of the center of the macula can serve as a proxy for detecting DME; hence, grades 0 and 1 are equivalent to non-DME and grade 2 is equivalent to DME in our classification scheme.

#### Messidor-2

The Messidor-2 (26, 27) dataset, as an extension of the Messidor-1 dataset, contained 1,748 (1,744 annotated as gradable) fundus images. In this study, we used 1,744 graded fundus images from the annotated Messidor-2 dataset by Krause et al. (28).

### Deep Learning Models

#### Fusion Model Network

**Figure 3:**
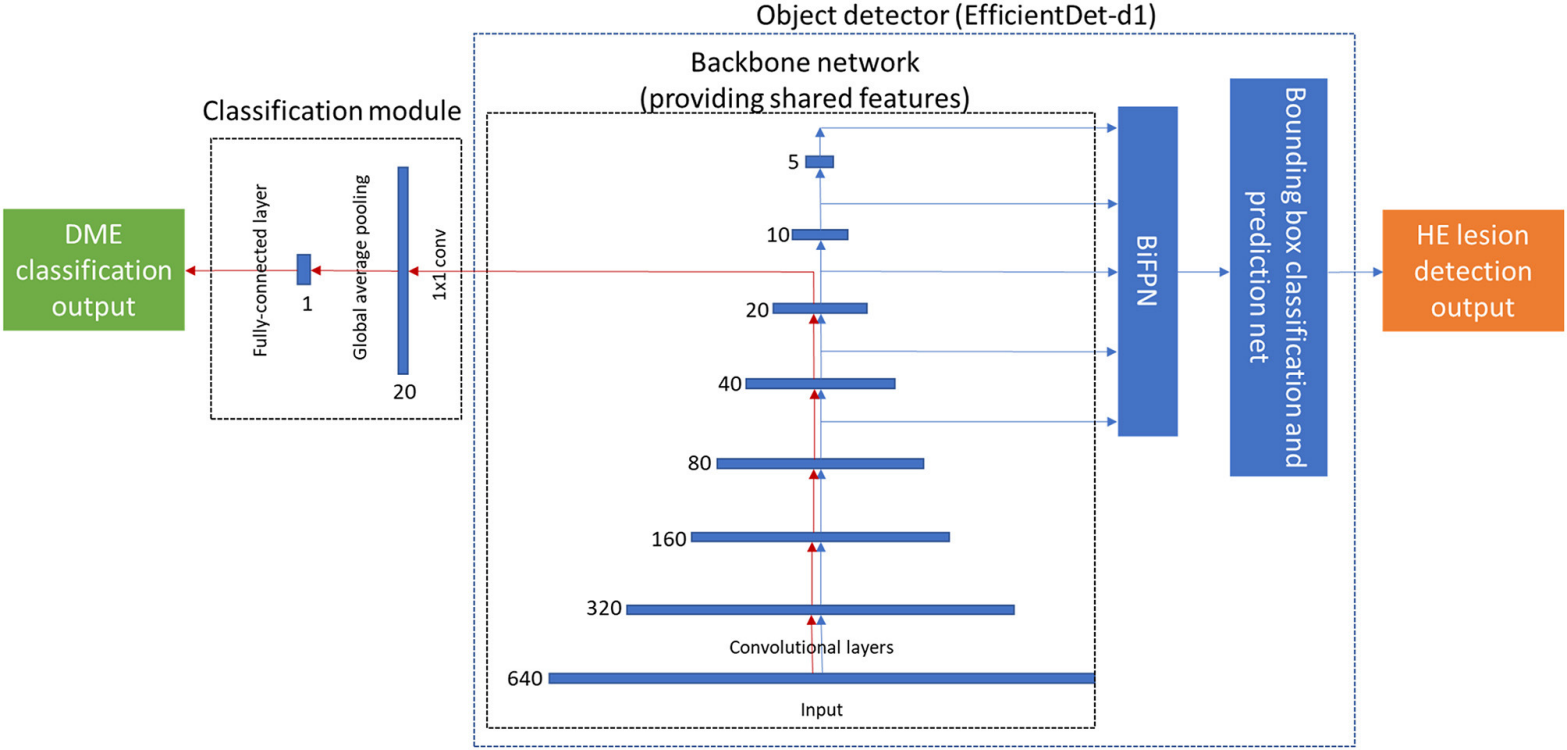
The architecture of the proposed end-to-end deep fusion model. The red arrow denotes the classification path, which forms the same architecture as EfficientNet-b1. The blue arrow denotes the lesion detection path, which has the same architecture as EfficientDet-d1. The number (640, 320, 160, …) near each feature map denotes its resolution.

We use EfficientDet-d1 (29) as the object detector because of its great balance between performance and resource usage. Because EfficientDet-d1 employs the feature extraction part of EfficientNet-b1 (30), we can readily use this aspect as the backbone in the fusion model. Lesion detection was implemented using bi-directional feature pyramid network (BiFPN). The classification module consisted of three layers and included a convolutional layer, a global average pooling layer, and a fully connected (FC) layer. The architecture of the fusion model is shown in Figure 3.

The fusion model is computationally efficient, as only one convolution layer is needed to extract higher-level features based on the output features obtained from the EfficientDet-d1 backbone. We denote *E*_*ob*_ as the loss function of EfficientDet-d1, *E*_*cl*_ as the loss function of the classification module, and the loss function for the fusion model is given by Equation (1), where ω_*ob*_>0 and ω_*cl*_>0, which are hyperparameters used to linearly combine the loss functions of the object detector and classifier.


(1) $$\begin{array}{l} E_{loss}=\omega_{ob}\times E_{ob}+\omega_{cl}\times E_{cl}\\ \;=\omega_{ob}\times E_{ob}-\omega_{cl}[\alpha {\left(1-p_{t}\right)}^{\gamma }\log(p_{t})] \end{array}$$

First, we use the equal weights for ω_*ob*_ and ω_*cl*_ in the initial training. Then analyzing the loss value obtained from the object detection model and the classification model. Second, we use the weighting factor (ω_*ob*_ and ω_*cl*_) that is inversely proportional to the loss value of the classifier or object detector to balance the loss, respectively. Finally, we retrain the fusion model using ω_*ob*_ (= 0.5) and ω_*cl*_ (= 100) to balance the loss obtained from both models, and avoid overfitting in the classification model or the object detection model. Our results showed that the setting ω_*ob*_=0.5 and ω_*cl*_=100 achieved a satisfactory balance. The parameters α ≥ 0 and γ ≥ 0 were also heuristically set to address the large class imbalance encountered during training. In general, α, the weight assigned to the rare class, should be slightly reduced as γ is increased (31). Here we used γ = 2, α = 0.25 as a default setting. The variable *p*_*t*_ is defined in Equation (2), where *p* is the estimated probability for the binary classification.


(2) $$p_{t}=\{\begin{array}{c} p\;if\;DME\;is\;the\;class\;label\\ 1-p\;otherwise \end{array}$$

#### Dual Model Approach

For comparison with the fusion model, we implemented a dual model, which consisted of two separate models including an image classifier and an object detector. The two separate models were trained and inferred separately. We used EfficientNet-b1 and EfficientDet-d1 as the image classifier and object detector, respectively, in our dual model for a fair comparison. EfficientNet stacked basic fixed modules and adjusted some hyperparameters such as the number of layers, number of channels, and input image resolution, using a neural architecture search. In addition, EfficientNet achieved state-of-the-art performance on ImageNet without using additional data.

### Device and Hyperparameters

All images of private dataset, Messidor-1, and Messidor-2 dataset were preprocessed before feeding our model. Each image was cropped to the fundus image with minimal black region (Supplementary Figure 1) and saved in the JPG image format. These cropped images were resized to input image sizes of 640 × 640 pixels. For image augmentation, we randomly flipped the images of private dataset vertically or horizontally. We trained and tested the model on an Intel Xeon E5-2660 v4 computer with 396 GB DRAM and NVIDIA Tesla V100 GPU using PyTorch with an initial learning rate of 0.0001, a dropout rate of 0.2 and a batch size of 16, for both the fusion and dual models. AdamW optimizer was used in the fusion model and dual model (EfficientDet-d1). Adam optimizer was used in the dual model (EfficientNet-b1). Values of weight decay of the fusion model, dual model (EfficientNet-b1), and dual model (EfficientDet-d1) were 0.01, 0.00001, and 0.01, respectively. Based on the setting of dropout and weight decay in the fusion model and dual model (EfficientNet-b1), the loss curves showed without overfitting in training and validation loss (Supplementary Figure 2).

To validate the feasibility of deploying our fusion model on an edge device, it was implemented on NVIDIA Jetson Xavier NX with 8GB of memory using PyTorch.

### Statistical Analyses

For the evaluation of performance in DME classification, we used metrics of sensitivity, specificity, accuracy, and area under the receiver operating characteristic curve (AUC). All metrics were listed with 95% confidence intervals (CIs). Receiver operating characteristic (ROC) curves were used to illustrate the overall performance using different cutoffs to distinguish between non-DME and DME. A two-proportion *z*-test was used to compare the two observed proportions obtained from the two models. The DeLong test (32) was used to compare the AUCs. Statistical significance was set at *p* < 0.05. In addition, we evaluated the performance of lesion detection according to Tseng et al. (20).

## Results

We trained the fusion model and dual model using the private dataset, and the performance was compared in three aspects: memory usage and execution time, DME classification, and HE detection.

### Memory Usage and Execution Time

We investigated the demand for memory and the execution time of the fusion and dual models to process one image from the private testing dataset. We used a command-line utility tool (Nvidia-smi) to evaluate the requirement of memory usage of the fusion model and the dual model to process one fundus image. In addition, the required time of processing one fundus image was calculated by using Python code “time.time()”. Table 3 shows that the fusion model required 1.6 GB of memory, whereas the dual model required 3.6 GB of memory. The mean required time of the fusion and dual model were 2.8 ± 1.5 s and 4.5 ± 1.8 s, respectively. This was averaged over the full testing dataset. These results show that the fusion model reduced the requirement for memory usage and execution time compared to the dual model.

**Table 3:** The data of memory usage and execution time for the fusion and the dual models to process one image of the private testing dataset.

**Resource consumption**	**Fusion Model**	**Dual model**
Memory (RAM)	1.6 GB	3.6 GB
Time (mean ± standard deviation)	2.8 ± 1.5 s	4.5 ± 1.8 s

### DME Classification

**Figure 4:**
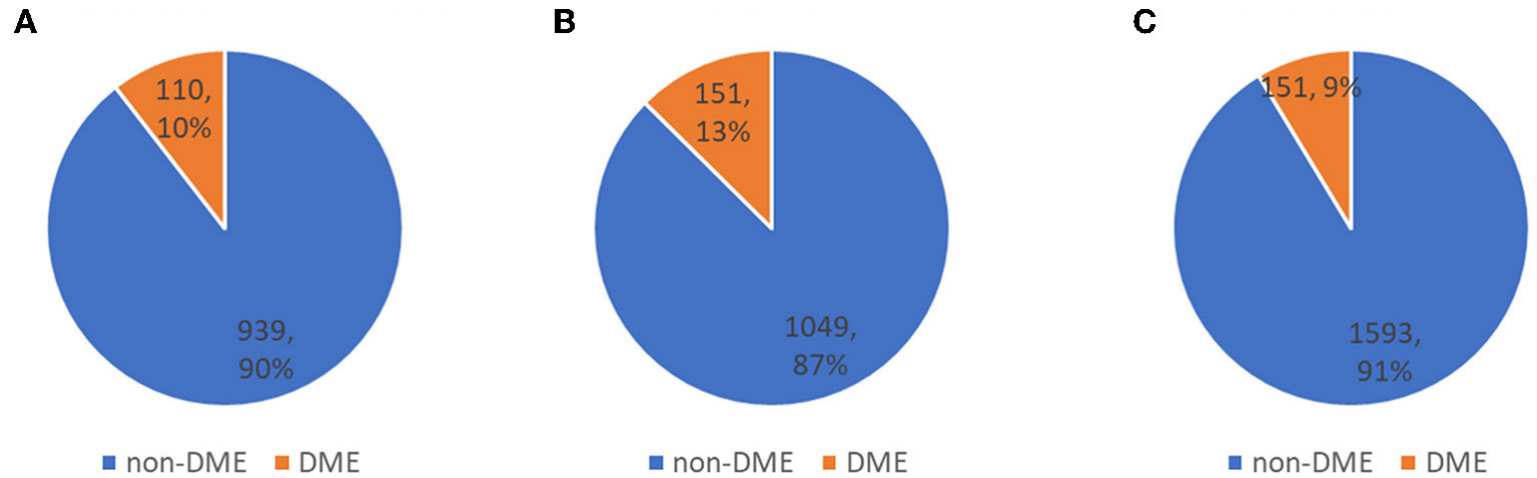
Distribution of three testing datasets. **(A)** private testing dataset, **(B)** Messidor-1, and **(C)** Messidor-2, used to evaluate classification performance.

**Figure 5:**
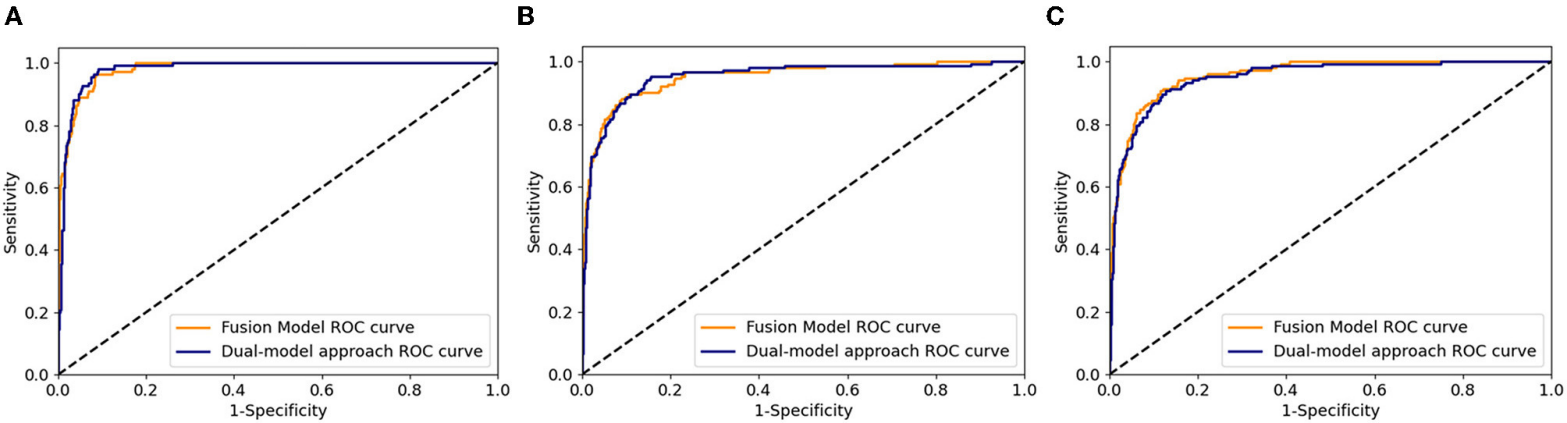
Receiver operating characteristic curves of fusion and dual model for the three datasets. **(A)** private testing dataset, **(B)** Messidor-1, and **(C)** Messidor-2.

The distribution of DME in a private dataset and two open datasets (Messidor-1 and Messidor-2) are shown in Figure 4. In Table 4, the performance of the fusion and dual models was evaluated using the AUC, sensitivity, specificity, and accuracy. The AUCs of both models were compared using the DeLong test for the three datasets. The result showed that there was no statistically significant difference between the models (*p*-values of 0.743, 0.942, and 0.114 for the private testing dataset, Messidor-1, and Messidor-2, respectively). Correspondingly, Figure 5 shows the results of the receiver operating characteristic curves (ROC) of both models for the three datasets. This result demonstrates that the performance of the fusion model is similar to that of the dual model.

**Table 4:** Performance of dual and fusion model for the three datasets.

	**Dataset**	**AUC (%)** **(95% CI)**	**Sensitivity (%)** **(95% CI)**	**Specificity (%)** **(95% CI)**	**Accuracy (%)** **(95% CI)**
Fusion model	Private testing dataset	98.1 (97.3, 98.9)	96.4 (92.9, 99.9)	90.1 (88.2, 92.0)	90.8 (89.1, 92.5)
	Messidor-1	95.2 (93.3, 97.1)	88.7 (83.7, 93.7)	90.2 (88.4, 92.0)	90.0 (88.3, 91.7)
	Messidor-2	95.8 (94.5, 97.1)	87.4 (82.1, 92.7)	90.2 (88.7, 91.7)	89.9 (88.5, 91.3)
Dual model	Private testing dataset	98.0 (97.2, 98.8)	96.4 (92.9, 99.9)	91.8 (90.0, 93.6)	92.3 (90.7, 93.9)
(EfficientNet-b1)	Messidor-1	95.2 (93.2, 97.2)	85.4 (79.8, 91.0)	91.7 (90.0, 93.4)	90.9 (89.3, 92.5)
	Messidor-2	95.1 (93.5, 96.7)	80.8 (74.5, 87.1)	92.7 (91.4, 94.0)	91.7 (90.4, 93.0)

### HE Lesion Detection

**Figure 6:**
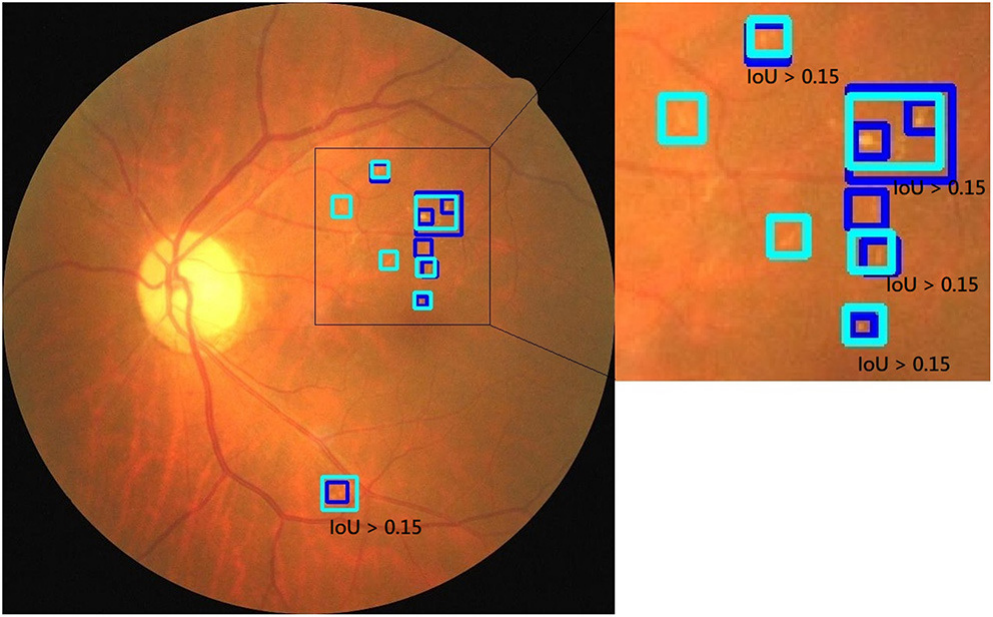
An example image with predicted and GT bounding boxes. The light blue boxes are the prediction result and the dark blue boxes are the GT result. Examples of IoU > 0.15 are marked in image.

**Figure 7:**
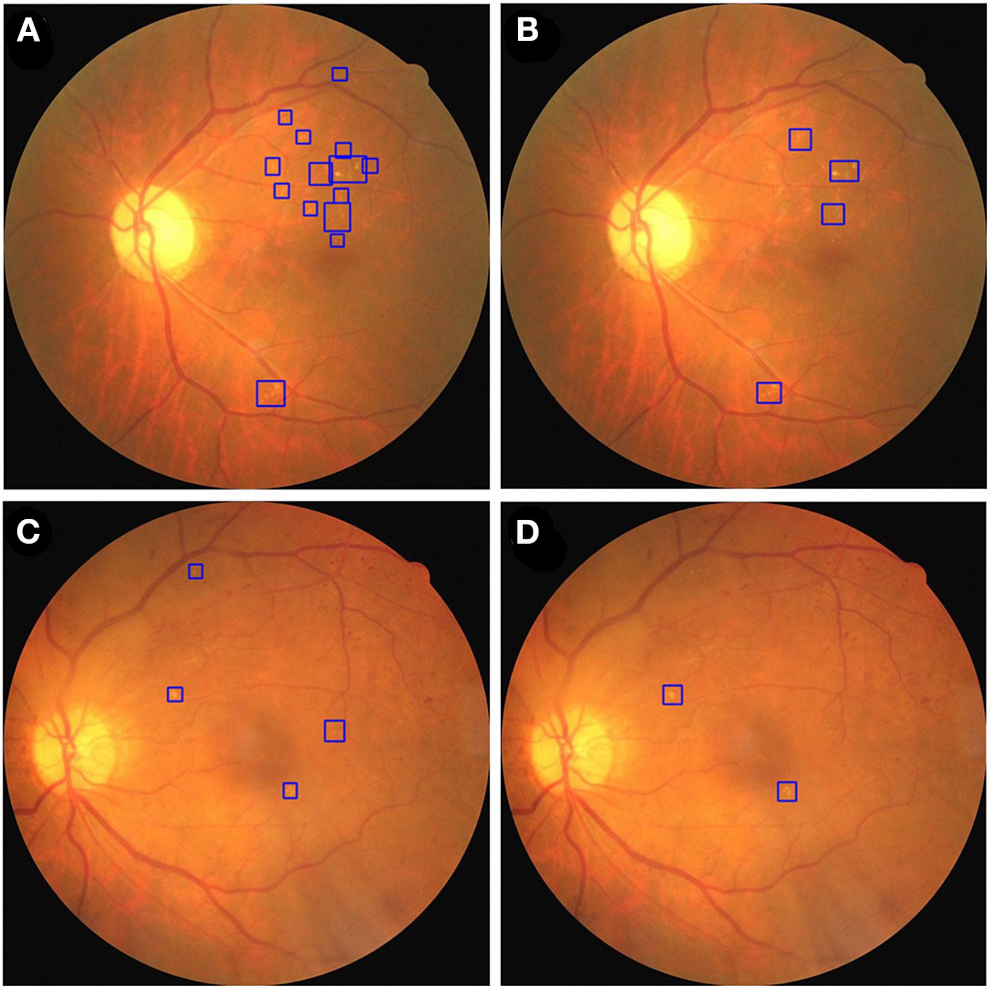
The fusion and dual model annotate HE lesions of two fundus images from the private testing dataset. A fundus image was annotated by fusion model **(A)** and dual model **(B)**. A second fundus image was annotated by fusion model **(C)** and dual model **(D)**. HE lesions are identified using blue bounding boxes.

We used fusion and dual models to detect HE lesions on our private testing dataset. We evaluated the performance of these models by using true positive, false positive, true negative, and false negative to calculate the accuracy, sensitivity, and specificity. Note that in the HE lesion detection, a true positive image is defined as one of the predicted HE area having an IoU > 0.15 compared to the GT location (as shown in Figure 6); a true negative image is defined as both GT and prediction without any lesion detection; a false positive image is defined as GT without any lesion detection but with prediction; and a false negative image is defined as GT with at least one location but no prediction or any prediction location having an IoU ≤ 0.15. In Table 5, the results of our private testing dataset revealed that the sensitivity of the fusion model was higher than that of the dual model, and the difference was statistically significant (*p* = 0.048). In addition, the specificity and accuracy of both models were not significantly different (*p* = 0.433 and *p* = 0.998, respectively). This result indicated that the fusion model could detect images with HE lesions more accurately. Furthermore, for lesion visualization, our models could output fundus image with the annotated HE lesion, as shown in Figure 7.

**Table 5:** The performance of HE detection in dual and fusion models.

	**Sensitivity (%)** **(95% CI)**	**Specificity (%)** **(95% CI)**	**Accuracy (%)** **(95% CI)**
Fusion model	79.5 (75.4, 83.6)	87.7 (85.2, 90.2)	86.3 (84.2, 88.4)
Dual model (EfficientDet-d1)	73.0 (68.4, 77.6)	89.2 (86.9, 91.5)	86.4 (84.3, 88.5)

### Optic Disc and Macula Detection

**Figure 8:**
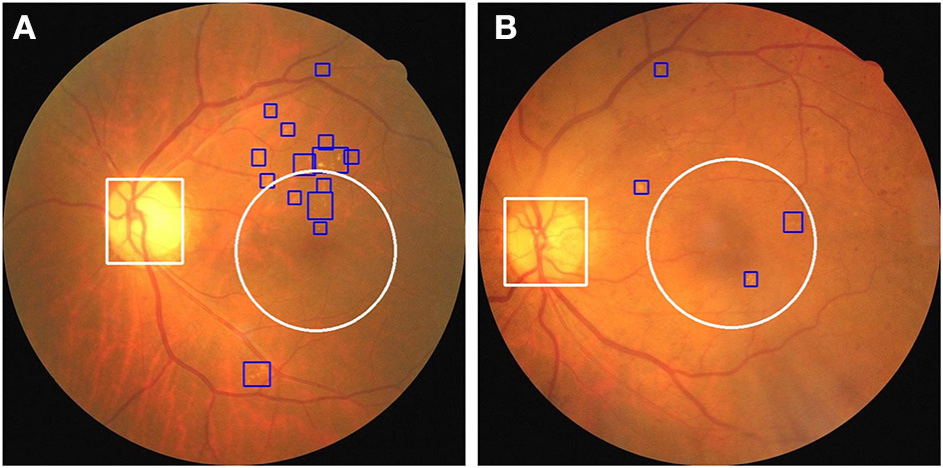
The integration of the visualization of the optic disc and the macula in two fundus images with annotated HE lesions. **(A)** Fundus image from Figure 7A annotated with optic disc and macula. **(B)** Fundus image from Figure 7C annotated with optic disc and macula. HE lesions are represented as blue bounding boxes. The white circle represents 1DD from the macula center. The white bounding box represents the optic disc.

Based on the preceding results, we established a novel end-to-end fusion model that can simultaneously facilitate disease classification and lesion detection. Clinically, anatomical landmarks such as the optic disc and the macula are examined by physicians to determine if there are HE lesions within 1DD from the center of the macula. Thus, we constructed an object detector to detect anatomical landmarks to facilitate advanced visualization. We trained an object detector using YOLOv3 (33) to detect the optic disc and macula. The details of the training process are provided in the Supplementary Material. The accuracy of the object detector for the detection of the optic disc and macula was 98.4 and 99.3%, respectively. Furthermore, the object detector could identify the optic disc using a white bounding box and an area within 1DD from the center of the macula using a white circle. These outlined boxes and circles can be integrated into the image results as shown in Figure 7. Figure 8 shows that physicians can instantly ascertain the presence of HE lesions within 1DD from the center of the macula, thereby enabling them to more reliably diagnose DME. Taken together, the results show that lesion visualization can more readily account for the result of DME classification when using the fusion model.

### Implementation on an Edge Device

To verify the feasibility of implementing the entire workflow on an edge device, we tested our fusion model and the anatomical landmark detector on NVIDIA Jetson Xavier NX with 8 GB of memory. The fusion model and the anatomical landmark detector required 7.4 ± 0.02 GB of memory and took 2.53 ± 0.72 s to infer a single fundus image on average. However, the combination of a dual model and an anatomical landmark detector cannot be implemented on edge devices owing to their memory constraints. In addition, we also tested the fusion model on DME classification of the three datasets and HE lesion detection using the NVIDIA Jetson Xavier NX with 8GB of memory. The performance for DME classification and HE lesion detection using the NVIDIA Jetson Xavier NX 8GB of memory was the same as that of the Intel Xeon E5-2660 v4 computer, as shown in Tables 4, 5, respectively.

## Discussion

**Figure 9:**
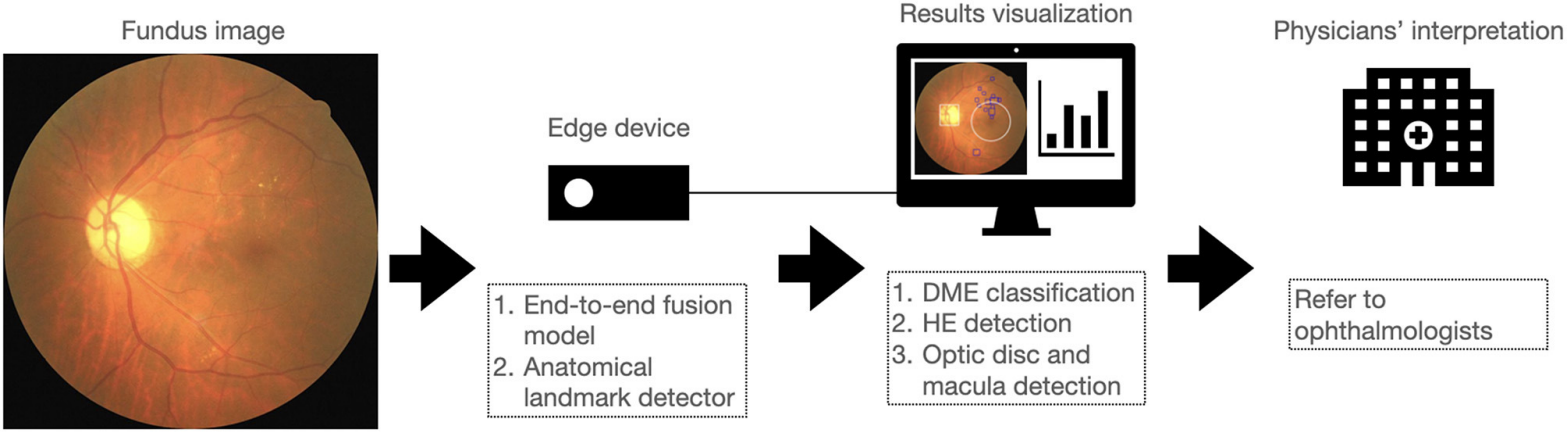
Overview of the proposed approach for implementing a system on an edge device that integrates DME classification, HE detection, and optic disc and macula detection to assist in the interpretation of fundus image by physicians.

In this study, we proposed a novel end-to-end fusion model to simultaneously facilitate DME classification and HE lesion detection. The performance of the fusion model for DME classification was similar to that of the dual model. The sensitivity of the fusion model for the detection of HE lesions was higher than that of the dual model. We further integrated the detection outputs from the fusion model and the anatomical landmark detector to improve lesion visualization. In addition, we implemented these two models on an edge device to facilitate portability and affordability in remote or resource-limited areas. As shown in Figure 9, we report for the first time the integration of the fusion model and a second object detector on an edge device for DME classification, HE detection, and optic disc and macula detection, for lesion visualization and improved interpretability of the AI model. This system allowed physicians not only to obtain the results of DME classification but also to observe the location of HE lesions related to the macula. This might assist physicians in assessing the necessity of referring diabetic patients to ophthalmologists for further examination and treatment.

Recently, several studies have used AI to classify DR with DME or DME only in the Messidor-1 and Messidor-2 datasets (16–19, 34–37). In Messidor-1, Sahlsten et al. (18) proposed an approach based on the ensemble of CNNs with AUC of 95.3%, Sensitivity of 57.5%, Specificity of 99.5%, and Accuracy of 91.6% to detect referable DME. Singh et al. (19) used a hierarchical two-stage ensemble CNN with Sensitivity of 94.7%, Specificity of 97.2%, and Accuracy of 95.5% to grade severity of DME. Ramachandran et al. (34) used a deep neural network software to detect referable DR (moderate DR or DME) achieving AUC of 98.0%, Sensitivity of 96.0%, and Specificity of 90.0%. Li et al. (35) used a cross-disease attention network with AUC of 92.4%, Sensitivity of 70.8%, and Accuracy of 91.2% to jointly grade DR and DME. In Messidor-2, Gulshan et al. (17) used inception-v3 architecture with AUC of 99.0%, Sensitivity of 87.0%, and Specificity of 98.5%. to detect referable DR. Abramoff et al. (16) used the IDx-DR 2.1 device to screen referable DR achieving AUC of 98.0%, Sensitivity of 96.8%, and Specificity of 87.0%. Yaqoob et al. (36) modified ResNet-50 architecture to screen referable DME achieving Accuracy of 96.0%. In 2021, Li et al. (37) used an improved inception-v4 with AUC of 91.7% to detect referable DME. Compared to the performances of above studies in the Messidor-1 and Messidor-2 datasets, the performance of fusion model was AUC of 95.2 and 95.8%, Sensitivity of 88.7 and 87.4%, Specificity of 90.2 and 90.2%, and Accuracy of 90.0% and 89.9% in the Messidor-1 and Messidor-2 datasets. In this study, the classifier of the fusion model was constructed by integrating the EfficientDet-d1 backbone and a classification module. This classifier had the same architecture as EfficientNet-b1. It was determined that the performance of DME classification was similar to that of the original EfficientNet-b1 in the dual model.

In fundus imaging, the determination of the presence and location of HE is useful for physicians in the diagnosis of DME. Several studies have used deep learning to detect HE lesions. Son et al. (38) used a class activation map (CAM) to generate a heatmap to identify the areas that contributed most to the model's decision in classifying DR and other ocular abnormalities. Lam et al. (39) used a sliding window to scan images and a CNN to detect whether HE lesions were present. In addition, Kurilová et al. (40) used the object detector of Faster-RCNN to detect HE lesions in fundus images. In this study, the object detector of our fusion model was modified from EfficientDet-d1, in which the backbone was co-used with the classification module during both the training and inference phases. We found that the performance of EfficientDet-d1 had significantly higher sensitivity for the detection of HEs compared to the original EfficientDet-d1 in the dual model. The higher sensitivity might be because the classification and object detection tasks are complementary in our system.

**Figure 10:**
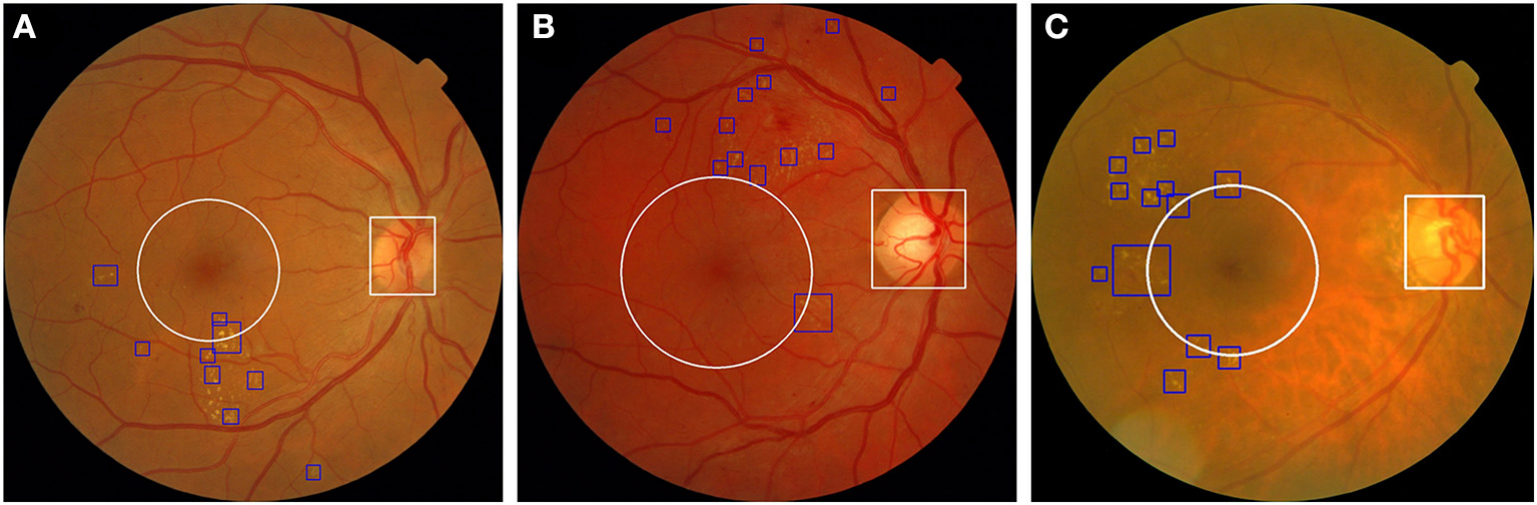
Three fundus images from the open dataset were classified as DME in our system. **(A)** Fundus image from Messidor-1 dataset. **(B,C)** Two fundus images from the Messidor-2 dataset. Our system labeled HE lesions, optic disc, and 1DD from the macula center. The blue bounding box represents the HE. The white circle represents 1DD from the macula center. The white bounding box represents the optic disc.

Typical deep learning models usually lack interpretability, whereas visualization is useful for physicians to assess the result of DME classification by AI. To resolve this problem, we trained another object detector, YOLOv3, to detect anatomical landmarks (optic disc and macula). Our system integrated the fusion model and another object detector to achieve visualization and increase the interpretability of the AI. We also applied this system to fundus images obtained from open datasets to examine its effect. As shown in Figure 10, three fundus images were classified as DME by our system, and it was possible to detect and annotate HE lesions, the optic disc, and 1DD from the macula center. These output fundus images can increase the interpretability of AI results for physicians.

Deep learning models often require large memory usage and computing power. It is difficult to deploy deep learning models on high-end computers in remote areas where resources are limited. Typically, edge devices or cloud computing is utilized to address this issue. However, cloud computing requires network resources. In some remote areas, there was no well-internet service to support cloud computing. Beede et al. (41) discovered that 2 h were required to screen ten diabetic patients using their cloud eye-screening system deployed in Thailand due to sluggish Internet service. Although the edge device is portable and does not require network connections, its small memory size and limited computing power are the primary hindrances. Singh and Kolekar (42) reduced the model size to resolve the storage issue associated with edge devices to classify COVID-19 using computed tomography scans of the chest. In our fusion model, the classifier and object detector co-used the backbone of the object detector. This design reduced the demand for memory usage and the execution time, as shown in Table 3. This fusion model is computationally efficient and can be deployed on an edge device with an anatomical landmark detector. In addition, due to traditional fundus camera without appropriate hardware (at least equipped with NVIDIA GeForce GTX 1070 8GB memory), one model to process the data on an edge device could resolve this issue. Therefore, this is the reason why we need to design a deep learning model to process the data in an edge device. Nonetheless, if the computer associated with the fundus camera has appropriate hardware, our model also could integrate into the computer system of camera without needing on an independent edge device.

Our study has several strengths. First, we used a large number of fundus images to train the model. Second, our model yielded satisfactory results for private and open datasets. The model could be implemented on fundus images for different ethnicities. Third, this system facilitates DME classification and the visualization of HE lesions, optic disc, and the macula. Therefore, it is expected that non-ophthalmologist physicians would have more confidence in DME diagnosis determined using AI. Fourth, this system can be deployed on an edge device. This device is portable and affordable. Thus, the proposed system could be applied to diabetic patients in remote or resource-limited areas.

This study has several limitations. First, drusen and the partial features of silicone oil retention are similar to those of HEs. These types of features were not well-trained in our system owing to limited data. This could lead to a false-positive result for DME. Second, we did not integrate the fusion model and anatomical landmark detector into one fusion model. Third, some diseases, such as myelinated fiber layer and optic disc edema, presented blurred boundaries of the optic disc. These diseases could influence the detection of the optic disc and cause inaccurate visualization of 1DD from the macula center.

Based on the obtained results, our future work will involve the application of the proposed system to other object detectors with a backbone that was originally a CNN image classifier, followed by the integration of the fusion model and the anatomical landmark detector into one fusion model on an edge device. Furthermore, we will also train this system to classify the grade of DR and annotate the locations of hard exudates, hemorrhages, soft exudates, microaneurysms, the optic disc, and the macula. This system will grade DR and DME, as well as provide lesion visualization to increase the interpretability of the AI results for physicians.

In conclusion, our system combines a novel end-to-end fusion model with a second object detector to perform DME classification, HE detection, and anatomical localization. It can identify DME and elucidate the relationship between HE and the macula. The entire system can facilitate higher interpretability and serve as a clinical reference for physicians. In addition, it can be implemented on a portable edge device. Clinically, this AI system can be used during the regular examination of DR to improve the interpretation of fundus imaging in patients with DME.

## Data Availability Statement

The original contributions presented in the study are included in the article/Supplementary Material, further inquiries can be directed to the corresponding author/s.

## Author Contributions

T-YW and P-YW conceived the fusion model. T-YW, J-TL, and C-LC prepared the manuscript and generated figures and tables, and participated in the experiments involving the fusion model and the DME severity classification model. P-YW conducted the experiments involving the fusion model. S-YC conducted the experiments on the anatomical landmark detector. T-YW, J-TL, C-LC, Y-WL, Y-HC, and J-TC participated in technical discussions. C-LC, Y-HC, and J-TC collected and annotated the data. All authors reviewed the manuscript, contributed to the article and approved the submitted version.

## Funding

This research was supported by the Industrial Technology Research Institute (Grant Number: K367B82210).

## Conflict of Interest

The authors declare that the research was conducted in the absence of any commercial or financial relationships that could be construed as a potential conflict of interest.

## Publisher's Note

All claims expressed in this article are solely those of the authors and do not necessarily represent those of their affiliated organizations, or those of the publisher, the editors and the reviewers. Any product that may be evaluated in this article, or claim that may be made by its manufacturer, is not guaranteed or endorsed by the publisher.
