# Supplementary material for: Diabetic Macular Edema Detection Using End-to-End Deep Fusion Model and Anatomical Landmark Visualization on an Edge Computing Device
Source: Front Med (Lausanne). 2022 Apr 4;9:851644. doi: 10.3389/fmed.2022.851644 (PMC9014123; doi:10.3389/fmed.2022.851644)
Supplement: Supplementary file 1 [file Data_Sheet_1.pdf]

Supplementary Material

1 Optic disc and macula detection

1.1 Dataset

We collected 347,042 fundus images that were acquired between 2007 and 2018 from 79,151 diabetic patients and three medical centers in Taiwan. All the images were acquired using a variety of fundus cameras with a 45° field of vision (FOV) and were anonymized for the retrospective aspect of the study. Low-quality images were excluded, and 101,145 fundus images from 51,042 diabetic patients were used for random sampling. Finally, a total of 36,487 fundus images from 25,856 patients were used to construct the detector. The study adhered to the tenets of the Declaration of Helsinki and was reviewed and approved by the institutional review board of the three medical centers: Tri-Service General Hospital (IRB: 1-107-05-039), Chung Shan Medical University Hospital (IRB: CSH: CS18087), and China Medical Hospital (IRB: CMUH10FREC3-062).

1.2 Annotations of dataset

Thirteen well-trained experts were recruited to annotate the fundus images. Each fundus image was annotated as a GT with an optic disc and a macula by one expert. Each annotation was represented using a bounding box format. The dataset was split into three sets: a training set, validation set, and testing set. Supplementary Table 1 lists the profiles of the three sets.

Supplementary Table 1. Dataset profile for the optic disc and macular detection tasks in this study.

	Training set	Validation set	Testing set
Number of images annotated using optic disc labels	31,532	2,747	949
Number of images annotated using macula labels	27,394	2,380	949
Total images	32,673	2,865	949

To evaluate the performance of optic disc and macula detection, we consider an image as "true positive" (TP) if it contains at least one predicted anatomical landmark that partially or completely overlaps with the GT anatomical landmark, and the intersection over union (IoU) value exceeds a pre-specified threshold. "True negative" (TN) indicates that no lesions are present for both GT and prediction. The accuracy is defined as (TP + TN)/(total number of images), and was listed with a 95%

confidence interval (CI). Here, we used 0.5 as the IoU threshold for evaluating the object detection model.

1.3 Visualization

The bounding box predicted by YOLOv3 with the highest confidence for each category (e.g., optic disc or macula) is reserved for visualization. In particular, for visualization of the macula, a white circle (Figure 8) is depicted to represent the area within 1DD from the center of the macula. The circle is defined by using the center coordinate of the bounding box of the macula as its center and the average length of the optic disc bounding box width and height as its radius.

1.4 Results

We evaluated the optic disc and macula detection using the testing dataset. This dataset only contains fundus images with a visible optic disc and the macula. Thus, there is no true negative, false positive, or specificity. In addition, the sensitivity and accuracy are the same. Here, we only list the accuracy of the optic disc, and the macula detection results in Supplementary Table 2. The object detector achieved accuracies of 98.4% and 99.3% for the optic disc and macula, respectively.

Supplementary Table 2. Performance for optic disc and macular detection in the testing set

	Accuracy (%) (95% CI)
Disc	98.4 (97.6, 99.2)
macula	99.3 (98.8, 99.8)

2 Supplementary Figures

2.1 Supplementary Figures

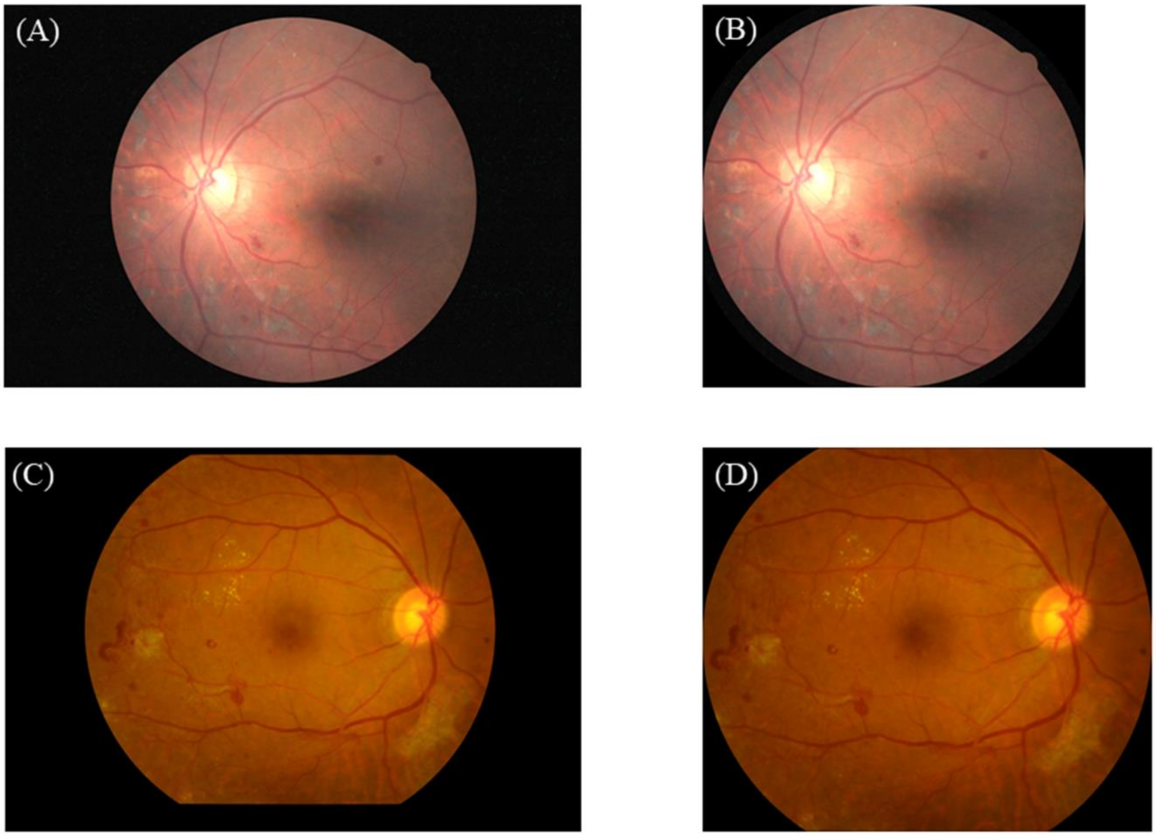

Supplementary Figure 1. The retinal photograph is cropped to the fundus image with minimal black region.

(A) and (B): Original images. (C) and (D): Images after cropped.

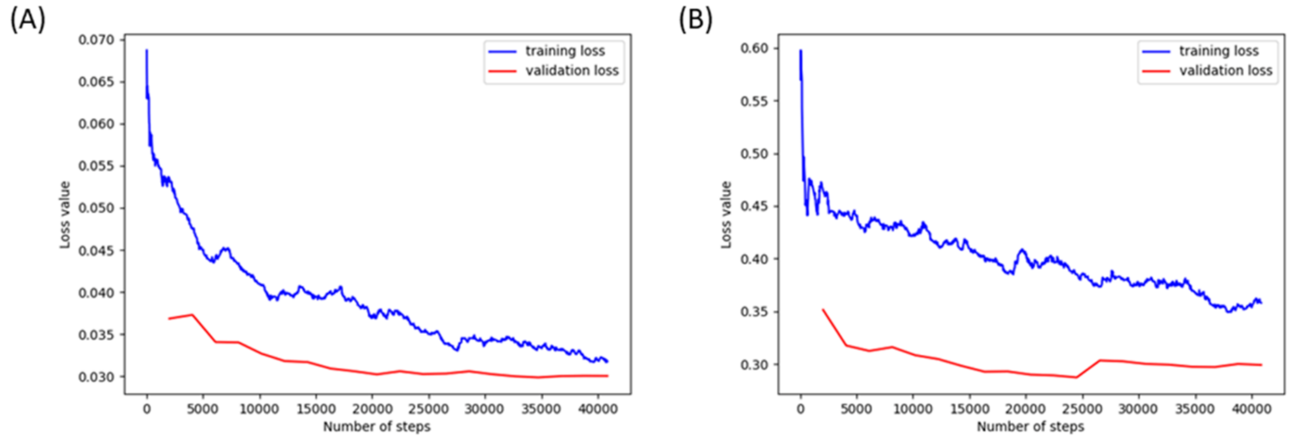

Supplementary Figure 2. Training and validation loss curves were exported from Tensorboard. (A) Fusion model. (B) Dual model (EfficientNet-b1).
